# Editorial: Modern treatment of autoinflammatory diseases

**DOI:** 10.3389/fmed.2024.1411940

**Published:** 2024-05-16

**Authors:** Maria Maslinska, Andra Balanescu

**Affiliations:** ^1^Early Arthritis Clinic, National Institute of Geriatrics, Rheumatology and Rehabilitation, Warsaw, Poland; ^2^Department of Internal Medicine and Rheumatology, Carol Davila University of Medicine and Pharmacy, Bucharest, Romania

**Keywords:** autoinflammatory diseases, treatment, current approach, rare diseases, immune system

As the series: “*Modern treatment of autoinflammatory diseases*” concludes, it is still far from exhausting its subject, in spite of presenting some of its very intriguing aspects.

In the series, we've paid closer attention to Still's disease, an example of autoinflammatory phenomena among rheumatic diseases (1) and—in the form of adult onset Still's disease (AOSD)—a possible mask of a developing malignancy, especially of lymphoproliferative diseases (2). Its potential therapies focus on the activity of proinflammatory cytokines, such as interleukin-1 (IL-1) and IL-6 (3).

A retrospective study using data from the “Autoinflammatory Disease Alliance” (AIDA) registry entitled: “*Efficacy of canakinumab in patients with Still's disease across different lines of biologic therapy: real-life data from the International AIDA Network Registry for Still's Disease*” by Vitale et al. presented the usefulness of anti-IL-1 treatment regardless of which line of treatment this biologic is used in.

Although it's not uncommon (5–12%), lung involvement is often overlooked in AOSD. Importantly, systemic juvenile idiopathic arthritis- lung disease (sJIA-LD) should be distinguished from the systemic form of JIA (4). Interestingly, an anaphylactoid reaction to drugs, including anti-IL-1 and anti-IL-6 monoclonal antibodies, is reported as a risk factor for the development of sJIA-LD (5–8).

The lung involvement in AOSD, associated with higher mortality (5), is indicated by BAL-F results, often with neutrophilia and by a picture of inflammatory infiltrates and consolidations in HRCT (7, 8)—although infection needs to be excluded. The authors Nies et al. of “*Rare, rarer, lung involvement in adult-onset Still's disease: a mini-review*” emphasize the importance of HRCT imaging in this condition and a significance of the increase in pro-inflammatory cytokines and acute phase proteins (IL1, IL-6, ferritin), which—although not specific to the lung involvement itself—correlate with its severity and a risk of developing acute respiratory distress syndrome (ARDS) and hemophagocytic lymphohistiocytosis (HLH)/macrophage activation syndrome (MAS), which may increase mortality. In treatment, GCs and methotrexate, calcineurin inhibitors, and in the case of recurrent disease, inhibition of IL-6 and IL-1 are used.

Infections, triggering factors of autoimmune and autoinflammatory diseases, are associated with a macrophage activation (6, 9) resulting in the presence of antibodies blocking INF-γ, STAT-1 phosphorylation and chemokine activity. These phenomena influence susceptibility to opportunistic infections in AOSD, as demonstrated in the study “*High-titer anti-interferon-*γ *neutralizing autoantibodies linked to opportunistic infections in patients with adult-onset Still's disease*” by Chen et al..

The treatment of autoinflammatory diseases has seen an increasing role of IL-1 inhibition. Canakinumab, playing currently the most prominent role in this regard, was the subject of the article titled: “*The safety and efficacy of canakinumab treatment for undifferentiated autoinflammatory diseases: the data of retrospective cohort two-centered study*” by Alexeeva et al., which focused on the analysis of young patients with undifferentiated autoinflammatory disease (uAID), showing that the majority (84%) of uAID patients in the treatment group presented remission.

Interestingly, the earlier CANTOS study conducted in patients after acute coronary syndromes and strokes with increased ultra-sensitive CRP, showed up to 15% reduction in deaths with the use of canakinumab. However, the risk of complications caused by infections was slightly higher (although overall low) in the canakinumab group compared to placebo, while in the longer term, there was a reduction in the incidence of lung cancer and mortality in the canakinumab group compared to placebo (10).

An another example of the effectiveness of inhibiting the pro-inflammatory activity of IL-1 was presented in the study “*Effectiveness and safety of anakinra in gouty arthritis: a case series and review of the literature*” by Jeria-Navarro et al., based on data from the literature regarding 551 patients treated both during flares and chronically, supporting the use of such treatment in refractory gout.

In cases of rare diseases, including mono- and polygenic autoinflammatory ones, studies of individual cases (often later taken into account for meta-analyses) play a vital role, especially when transcriptomic analysis is taken into account (9). Hua et al. in the article “*Single-cell transcriptomic analysis in two patients with rare systemic autoinflammatory diseases treated with anti-TNF therapy*” focused on the analysis of the mechanism of action of anti-TNF treatment in autoinflammatory disease via the interferon response pathway and inhibition of macrophage differentiation, showing that the introduction of single-cell RNA sequencing technology to rare and new diseases may become increasingly important.

Still, in observational research analysis of large numbers of cases remains essential—as demonstrated in the study from AIDA registry of the very rare disease: periodic fever, aphthous stomatitis, pharyngitis, and cervical adenitis (PFAPA), where medical data from 85 patients with this disease were retrospectively analyzed to demonstrate the effectiveness of using *Streptococcus salivarius* K12 (SsK12) to inhibit opportunistic and enrich commensal flora in patients with PFAPA syndrome (“*Preliminary data revealing efficacy of Streptococcus salivarius K12 (SSK12) in Periodic Fever, Aphthous stomatitis, Pharyngitis, and cervical Adenitis (PFAPA) syndrome: a multicenter study from the AIDA Network PFAPA syndrome registry*” by La Torre et al.). It's noteworthy that a prospective, randomized clinical trial investigating the use of the SsK12 probiotic in oral mucositis among patients undergoing head and neck radiotherapy for malignant tumors demonstrated symptom alleviation and a decrease in mucositis occurrence, alongside a favorable safety profile (11).

To sum up, progress in treating autoinflammatory and autoimmune diseases is related to the constantly expanding knowledge about the pathogenesis of these diseases. The treatment targeted at specific cytokines, such as e.g., IL-1, and other elements of the immune system involved in the activation of inflammation, is currently therapeutic standards.

## Author contributions

MM: Writing—original draft, Writing—review & editing. AB: Writing—original draft, Writing—review & editing.
